# Molecular Methodologies for Improved Polymicrobial Sepsis Diagnosis

**DOI:** 10.3390/ijms23094484

**Published:** 2022-04-19

**Authors:** Mariam Doualeh, Matthew Payne, Edward Litton, Edward Raby, Andrew Currie

**Affiliations:** 1 Centre for Molecular Medicine & Innovative Therapeutics, Murdoch University, Murdoch, WA 6150, Australia; mariam.doualeh@murdoch.edu.au; 2 Wesfarmers Centre of Vaccines and Infectious Diseases, Telethon Kids Institute, Perth, WA 6009, Australia; 3 Women and Infants Research Foundation, Perth, WA 6008, Australia; matthew.payne@uwa.edu.au; 4 Division of Obstetrics and Gynaecology, University of Western Australia, Perth, WA 6008, Australia; 5 Intensive Care Unit, Fiona Stanley Hospital, Murdoch, WA 6150, Australia; ed.litton@health.wa.gov.au; 6 Intensive Care Unit, St. John of God Hospital, Subiaco, WA 6009, Australia; 7 State Burns Unit, Fiona Stanley Hospital, Murdoch, WA 6150, Australia; edward.raby@health.wa.gov.au; 8 Microbiology Department, Path West Laboratory Medicine, Murdoch, WA 6150, Australia

**Keywords:** polymicrobial sepsis, molecular diagnostics, PCR, pathogen interactions

## Abstract

Polymicrobial sepsis is associated with worse patient outcomes than monomicrobial sepsis. Routinely used culture-dependent microbiological diagnostic techniques have low sensitivity, often leading to missed identification of all causative organisms. To overcome these limitations, culture-independent methods incorporating advanced molecular technologies have recently been explored. However, contamination, assay inhibition and interference from host DNA are issues that must be addressed before these methods can be relied on for routine clinical use. While the host component of the complex sepsis host–pathogen interplay is well described, less is known about the pathogen’s role, including pathogen–pathogen interactions in polymicrobial sepsis. This review highlights the clinical significance of polymicrobial sepsis and addresses how promising alternative molecular microbiology methods can be improved to detect polymicrobial infections. It also discusses how the application of shotgun metagenomics can be used to uncover pathogen/pathogen interactions in polymicrobial sepsis cases and their potential role in the clinical course of this condition.

## 1. Introduction

Sepsis is a dysregulated host response to infection resulting in organ dysfunction [1]. The annual global incidence of sepsis is estimated at approximately 49 million cases with 11 million deaths worldwide [2]. Suboptimal diagnostic modalities can lead to delayed targeted antimicrobial therapy, contributing to high mortality [3,4]. Early treatment with narrow-spectrum antibiotics requires rapid identification of pathogens, which is often not possible with current microbiological methods that rely on blood culture as the gold standard [5]. In addition to slow reporting times, blood culture often misses detection of one or more potential pathogens, with culture-independent next-generation sequencing technologies demonstrating that polymicrobial sepsis scenarios may occur more frequently than historically recognised [6,7,8]. However, most available epidemiological studies on polymicrobial sepsis to date are based on blood culture data, so while the current prevalence is estimated to be up to 14% [9,10], the true burden is likely underestimated.

Compared to monomicrobial sepsis, polymicrobial sepsis is associated with higher illness severity, higher progression rates to severe sepsis and septic shock, longer hospital stays and higher mortality rates in all age groups [3,4,11,12,13]. Factors that contribute to these differences may include variations in the anatomical source of infection, the relative proportion of immunocompromised hosts and the adequacy of first-line antimicrobial therapy [3,11,14,15]. Investigations of sepsis that employ integrative omics methodologies have demonstrated the complexity of host–pathogen interactions [16,17]. There is emerging evidence that microbe–microbe interactions also play a significant role in driving the host inflammatory response observed in polymicrobial infections [18,19]. In other polymicrobial diseases such as polymicrobial lung infections, in vitro interactions between organisms have been shown to enhance pathogen survival and increase the overall virulence exhibited by the detected microorganisms [18]. 

Molecular methods that can rapidly and accurately detect all pathogenic microbes contributing to the clinical syndrome of sepsis are urgently required. In addition, these methods can help to uncover the complex interactions between pathogens in polymicrobial sepsis and how this may affect the host response. The aim of this review is to provide an overview of the clinical significance and characteristics of polymicrobial sepsis and the limitations of the current diagnostic methods used to detect it. Potential molecular approaches to improve microbiological detection and develop more reliable diagnostic methods will be examined. The use of shotgun metagenomics to study the microbial metagenome in patient sepsis samples is also proposed as a means to define the role of pathogen–pathogen interactions in driving polymicrobial sepsis. 

## 2. Polymicrobial Sepsis

### 2.1. Definitions

The reported prevalence of polymicrobial sepsis ranges from 2 to 14% [9,14]. Although many factors contribute to this variation, such as cohort location and the diagnostic methods adopted, the lack of standard definitions makes it challenging to assess the true prevalence. There is no current standard definition of polymicrobial sepsis. However, based on the Sepsis-3 definition [1], it can be described as life-threatening organ dysfunction caused by a dysregulated host response to infection involving more than one pathogen. Polymicrobial sepsis will often be associated with polymicrobial bacteraemia. However, polymicrobial bacteraemia can be defined in different ways (Table 1). In general, studies adopting a more stringent definition (e.g., Definitions 1–3, Table 1) report lower incidences of polymicrobial bacteraemia (~2 to 11%) whereas those with the broadest definition (e.g., Definition 4, Table 1) report higher incidences of ~11 to 14% [3,9,11,14,20]. 

Consensus definitions for both polymicrobial bacteraemia and polymicrobial sepsis are needed to improve early recognition, clinical management and research quality. We propose that based on the current gold standard detection method, polymicrobial bacteraemia should be described as the isolation of more than one pathogen from one or more blood cultures collected within a 48 h period, as pathogens detected outside this time window are commonly separate monomicrobial hospital-acquired infections, rather than universally polymicrobial infection [21]. However, as the development of more sensitive molecular microbiology detection tools continues to advance, this may change to the detection of bacterial cell-associated nucleic acids from multiple pathogens in the same sample. Reaching a consensus definition for polymicrobial sepsis will be less straightforward. This is because the definition will need to take into account the microbial contribution to pathogenesis, an aspect that is neglected in the current definition of sepsis [1]. Extensive research is required to determine what this microbial contribution entails, and what the subsequent host response involves. 

Due to the limited number of recent studies on polymicrobial bacteraemia and polymicrobial sepsis, this article will review studies using all of the definitions listed in Table 1. Further, the term ‘bacteraemia’ will be used instead of ‘sepsis’ when studies do not confirm the sepsis status of participants.

### 2.2. Clinical Significance

Polymicrobial sepsis is associated with more than three times higher progression rates (26.7% vs. 7.8%) to severe sepsis and septic shock than monomicrobial sepsis [3]. Patients with polymicrobial sepsis are also less likely to receive correct empirical antimicrobial therapy especially if fungi are involved, with one study reporting only 34% of polymicrobial cases received correct empiric antimicrobial therapy compared to 59% of monomicrobial cases [4]. This is a significant risk factor for mortality, with Goldman et al. reporting a 3-day mortality rate of 8% for patients with adequate therapy compared to 57% for those without [14].

In general, mortality rates are significantly higher in patients with polymicrobial sepsis compared to those with monomicrobial sepsis. Pavlaki et al. reported mortality rates of 38% vs. 25% in adult hospitalised patients with polymicrobial sepsis or monomicrobial sepsis, respectively [11,22]. Short-term mortality (3-day mortality) was significantly higher in polymicrobial cases (16%) compared to monomicrobial controls (6%) in a study by Goldman et al. [14]. Mortality can be more than two-fold greater in neonates with polymicrobial bacteraemia (47%) compared with monomicrobial bacteraemia (20%) and appear even higher in immunocompromised adults [9,22]. For example, Fontana et al. reported a significantly increased likelihood of death (adjusted odds ratio 3.78 [95%CI 1.26 to 11.32]) in cancer patients with polymicrobial bacteraemia compared to those with monomicrobial bacteraemia [22]. However, not all studies show an association with higher mortality rates [3,23,24,25]. Tsai et al., for example, reported that neonates with polymicrobial bacteraemia presented with more severe sepsis and required more modification of antimicrobial therapy, but mortality rates were not significantly higher compared to those with monomicrobial bacteraemia [3].

### 2.3. Risk Factors for Developing Polymicrobial Sepsis

Immunosuppression resulting from surgery, burn injuries or febrile neutropenia have been recognised as significant risk factors for developing polymicrobial sepsis [15,26,27]. Additional risk factors that have been identified are listed in Table 2. 

### 2.4. Infection Sources

Due to the abundance of opportunistic pathogens in the gut microbiome, the gastrointestinal tract is one of the most common sources of polymicrobial sepsis, with approximately 15–34% of cases having an intraabdominal source [11,14,20,28,29]. Intraabdominal infections, short gut syndrome, biliary stenting and cholangitis are often reported in association with polymicrobial sepsis [11,14,28]. Similar organisms are implicated in ascending infection of the hepatobiliary tract, with more than a third of polymicrobial cases having a biliary source in one study [28]. Early recognition of the source, controlling the foci of infection and restoring optimal function of the infection site are critical for improving patient outcomes [30]. It is recommended that polymicrobial bacteraemia, especially due to combinations of Gram-negative bacilli, anaerobic bacteria and enterococci should always prompt urgent investigation for an intraabdominal infection [29,31]. 

Pneumonia in sepsis patients is also associated with high mortality, with reported mortality rates of 66.7% and 48.6% for hospital-acquired and ventilator-associated pneumonia, respectively [11,28]. Pneumonia is also increasingly recognised as a polymicrobial condition, with the contribution of multiple bacteria and viruses [32]. The main pathogens involved in hospital-acquired pneumonia polymicrobial bacteraemia include *Escherichia coli* and *Klebsiella pneumoniae* [11]. Grau et al. reported that 30% of polymicrobial pneumococcal bacteraemia involved *E. coli,* suggesting a gut origin [33], possibly involving alterations in the normal pharyngeal microbiota during hospitalisation, and particularly following antibiotic therapy [34]. Antibiotic therapy suppresses the growth of commensal microbes, such as streptococci, in the pharynx, and promotes replacement by Gram-negative bacilli [34] which can then promote polymicrobial infection following aspiration of oropharyngeal or gastric content [33].

Soft tissue infections are less common sources of polymicrobial sepsis, accounting for up to 11% of cases [14]. Bacteria isolated from polymicrobial soft tissue infections such as synergistic gangrene and type I necrotising fasciitis include combinations of Gram-positive cocci, Gram-negative bacilli and anaerobes e.g., *Bacteroides* sp. [35,36]. These infections can result from factors such as infected surgical wounds, burns and chronic skin ulcers, all of which can rapidly progress to polymicrobial sepsis [35,36]. While these severe soft tissue infections occur less frequently, they can be fatal if not recognised and treated aggressively [36,37].

### 2.5. Microbes Associated with Polymicrobial Sepsis

**Figure 1:**
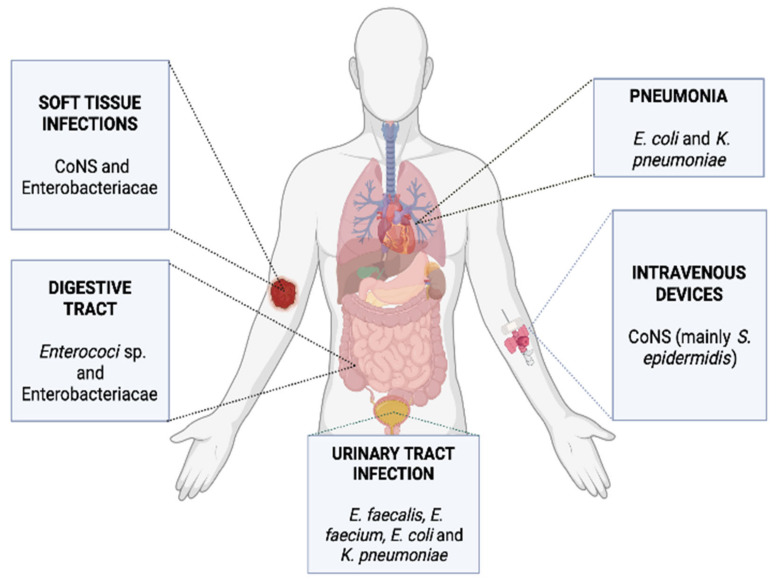
Most common microbes implicated in polymicrobial sepsis and their sources.

The most common microbes isolated from polymicrobial blood cultures are detailed in Table 3 and Figure 1. Some of these bacteria, such as coagulase-negative staphylococci (CoNS) occur frequently in neonates and children whereas others such as Enterobacteriaceae are more commonly isolated from adults [4,9,11,14]. Further, some pathogens are more adapted to drive polymicrobial infections than others [38,39]. Many of these bacteria possess virulence factors that aid the formation of biofilms, which may occur on medical devices, such as indwelling catheters and disperse into the bloodstream, leading to polymicrobial sepsis [40,41]. The establishment of biofilms confers a great advantage to microbes, as this can allow them to coordinate the utilisation of nutrients and increase resistance to antimicrobials, ultimately promoting dysregulation of the host’s immune response [38,39]. 

Interestingly, some organisms commonly involved in monomicrobial sepsis, such as *Staphylococcus aureus*, are not as frequently implicated in polymicrobial sepsis [42]. In one study, only 6% of all *S. aureus* cases were polymicrobial [43]. Typically, they co-infect with other Gram-positive aerobes and *Acinetobacter baumannii* [42,43,44,45]. In up to 10% of cases, they co-infect with fungi, which are also rare in polymicrobial sepsis [42,45]. Bouza et al. reported that only 7% of all polymicrobial sepsis cases involved *Candida* spp. However, these mixed staphylococcal–fungal infections can be devastating, with reports of higher ICU admission, longer hospital stays and higher mortality compared to both polymicrobial bacteraemia and monomicrobial fungal infections [45,46]. Similarly, it is well established that viral infections can predispose hosts to secondary bacterial infections, and that these co-infections are often worse than if the pathogens were to infect alone [47]. However, the prevalence of polymicrobial viral sepsis is unknown, and the lack of research into viral sepsis, in general, makes it challenging to calculate an estimate [48]. 

**Table 3:** Bacterial species that are commonly implicated in polymicrobial sepsis.

Pathogen	Common Co-Pathogens	Age Group/s	Virulence Factors
Enterococci*—*mainly *Enterococcus faecalis* and *Enterococcus faecium* [49,50]	Enterobacteriaceae, coagulase-negative staphylococci (CoNS) and *A. baumannii* [11,51]	All [3,4,9,14,28,52]	Gelatinase production to break down proteins (e.g., haemoglobin) and degrade fibrin, allowing bacteria to disseminate [53] Enterococcal surface protein (esp), facilitates adhesion to fibrinogen and collagen to promote biofilm formation [54,55]
*E. coli* [3,14,28]	Mainly other Enterobacteriaceae [11,28]	More common in adults [11,14,28]	Lipopolysaccharide [56], causes immune dysregulation and allows other organisms to colonise and co-infect [57]
*K. pneumoniae* [3,11,14,28]	Mainly other Enterobacteriaceae, particularly *E. coli* [58]	More common in adults [11,14,28]	Bacterial capsule for immune evasion, pili to facilitate adhesion, and siderophores to scavenge iron from the host [59,60]
CoNS—mainly *Staphylococcus epidermidis* [49,50],	Other CoNS species [10], Enterococci and *Candida* spp. [9]	More common in neonatal and paediatric bacteraemia [3,4,9,52]	Phenol-soluble modulins that increase biofilm complexity and facilitate bacterial dissemination [41,61], trigger strong pro-inflammatory responses via the production of IL-8 [62], and lead to the development of necrotising enterocolitis in neonates [63]

## 3. Detection of Polymicrobial Sepsis

### 3.1. Current State 

The current ‘gold standard’ method of detecting bloodstream infections is based on culture-dependent techniques [64]. The limitations of these have previously been discussed in detail by Dubourg et al., with the main ones being the slow turnaround times, low sensitivity and the restricted detection of only organisms that grow under laboratory culture conditions [64]. There are additional drawbacks when the infection is polymicrobial. Firstly, when bacteria are present at unequal densities or have different growth rates, growth of the more abundant or fast-growing organism can reach the automated blood culture system threshold for positivity, prompting sub-culture analysis for identification, while low-titre or slow-growing microbes may not have proliferated enough for subsequent detection [65]. For example, low-titre/slow-growing bacteria may be missed in Gram stains of positive cultures [3]. Tsai et al. reported that 11 out of 12 bacteraemia cases in neonates initially reported as Gram-negative based on preliminary Gram stain analysis were found to be mixed once final identification was completed, leading clinicians to incorrectly narrow antibiotic coverage in some cases [3]. Secondly, Matrix-Assisted Laser Desorption/Ionisation-Time of Flight (MALDI-TOF) analysis, which is employed in many routine labs for rapid pathogen identification, performs very poorly at detecting polymicrobial infections, often only reporting the predominant organism [64,66,67]. Studies have shown that all organisms are correctly identified in only 2–34% of polymicrobial blood cultures using MALDI-TOF [66,67]. Newer versions of MALDI-TOF, such as the updated Bruker MBT Sepsityper module, offer slight improvements, although sensitivity is hampered when more than two organisms are present [66]. These limitations of culture-based methods lead to significant inaccuracy and delays in the detection of all causative organisms in polymicrobial sepsis cases, further delaying the commencement of correct antimicrobial therapy [3]. Research into the development of more rapid and sensitive polymicrobial detection methods is therefore crucial.

### 3.2. Molecular Diagnostic Methods

Several reviews have analysed emerging and current culture-independent molecular methods for diagnosing bloodstream infections. Zhang et al. evaluated some of these technologies specifically for the detection of polymicrobial infections [68]. Compared to other technologies such as peptide nucleic acid–fluorescence in situ hybridisation, quantitative PCR (qPCR) was determined to be among the most rapid and cost-effective, exhibiting high sensitivity, adequate accuracy and multiplexing capacity [68]. The use of next-generation sequencing, for example, 16S ribosomal RNA (rRNA) gene sequencing or cell-free DNA sequencing, may also be feasible due to their high accuracy and multiplexing capabilities [68,69]. In the following section, the performance of current qPCR and DNA sequencing applications for the detection of polymicrobial sepsis will be briefly discussed. Possible solutions to commonly faced challenges, most of which stem from low blood volumes, low bacterial loads, high host DNA content and microbial DNA contamination from the environment and laboratory reagents will also be reviewed. 

#### 3.2.1. PCR on Positive Blood Cultures

PCR testing from positive blood cultures ensures sufficient amplification of starting inocula, thus increasing qPCR sensitivity compared to direct PCR of whole blood. One commercial PCR test performed on positive blood culture is the Cepheid Xpert MRSA/SA BC test, which is used to identify methicillin-resistant *S. aureus* (MRSA) and methicillin-susceptible *S. aureus* (MSSA) in approximately one hour [70]. The assay targets the *spa* gene, the *mecA* gene and the SCC*mec*–*orfX* junction, and is usually ordered when Gram-positive cocci in clusters are reported in Gram stains, to distinguish between *S. aureus* and CoNS [71]. The test has up to 100% sensitivity and specificity for MRSA detection [70,72]. Spencer et al. validated this test on paediatric samples and reported that even with polymicrobial samples containing *S. aureus* and additional organisms such as *E. coli* and *Micrococcus* sp., 100% sensitivity was achieved for both MSSA and MRSA [72]. However, as with most nucleic acid amplification tests, the results should be interpreted with caution, as amplification failures may occur due to target mutations not being taken into account in the testing panel [71]. For example, a 2018 report described two cases of misidentified *S. aureus* (one MSSA and one MRSA) due to deletions within the S region of the *spa* gene [71]. However, the overall rapid turnaround times and high sensitivity of the test can lead to better patient outcomes, such as shorter hospital stays and duration of IV antimicrobial administration [73]. One study showed a potential reduction in broad-range antibiotic therapy by 0.3 days per patient in 18% of cases using the test compared to routine culture [70].

The BioFire FilmArray Blood Culture Identification (BCID) panel is another commercial multiplex PCR system, targeting 24 pathogens (19 bacterial and 5 yeast) and 4 AMR genes (*mecA*, *VanA*, *VanB* and *BlakPC*). The newer version of this system, the BioFire FilmArray BCID2, is capable of detecting an additional nine pathogens and five AMR genes [74] but is yet to be specifically validated on polymicrobial samples. The BCID test generally exhibits high sensitivity and specificity (up to 96.5% and 99.7%, respectively) [75], although its performance at detecting polymicrobial infection varies. A recent study found that the test correctly identified all pathogens when two organisms were present in 91.5% of cases of simulated polymicrobial blood cultures, created by combining two positive blood cultures or by spiking *C. albicans* into positive blood cultures. However, this fell to <43% when three or more organisms were present [67]. Overall, the BCID panel can identify all pathogens in approximately 70% of polymicrobial cases and has been demonstrated to correctly identify a case of polymicrobial bacteraemia initially reported as monomicrobial using the routine method, which led to the rapid adjustment of antimicrobial therapy for the patient [76,77]. This application has also been shown to be beneficial in under-resourced remote laboratories, which typically refer patient samples to larger hospital laboratories equipped with routine technology, such as MALDI-TOF [78]. In these settings, turnaround times can be reduced by more than 24 h [78]. 

#### 3.2.2. PCR on Whole Blood

Performing qPCR directly on whole blood to identify sepsis pathogens has the advantage of delivering faster results, but in practice, it is associated with poor accuracy. The SeptiFast system (Roche) was the first commercial platform for whole blood testing. This system has previously been demonstrated to detect polymicrobial infections missed by routine culture, including a case in a preterm infant where only 0.5 mL of blood was required to detect DNA of additional bacteria and fungi, leading to the adjustment of antimicrobial therapy [79]. However, a meta-analysis of 54 studies compared the overall performance of this test with routine culture and estimated the sensitivity to be 68% and the specificity to be 86% [80]. Perhaps for this reason, the test was discontinued in 2019 [81]. The Magicplex Sepsis Real-Time Test is a currently available test with >90 targets, a runtime of 3–6 h and a LOD of 30 CFU/mL [82]. It is unclear how well this test performs at detecting polymicrobial infections, but the overall diagnostic potential is hampered by its very poor sensitivity (29–47%) [82,83]. Istanbullu et al. evaluated the performance of 16S rRNA gene qPCR using hydrolysis probes for the diagnosis of neonatal sepsis, and reported sensitivity and specificity of 16.6% and 97.8%, respectively [84]. However, it is important to note that many of these PCR tests have high negative predictive values (up to 95%), suggesting they would be more appropriate as screening tools to exclude infections, predict patient outcomes and monitor therapy rather than to identify pathogens [84,85]. For example, determining the bacterial load in blood has been shown to be a promising approach to identify patients with severe sepsis [86]. A 16S rRNA gene level greater than 1237 copies/mL has previously been associated with a high chance of developing severe sepsis [86]. 

One of the reasons why PCR performs poorly directly on whole blood is because it is challenging to concentrate bacterial cells, particularly when bacterial loads are as low as 10–100 CFU/mL, and in cases of low blood volume aspiration, such as with neonates, where a blood sample of only 0.5 mL may contain as low as 0.5–5 CFUs, resulting in lower detection [87,88,89,90]. Microfluidics-based methods can help separate bacterial cells from the dominant host cells and blood components based on cell size differences [87]. However, separation by size is difficult due to the size similarities of bacteria and red blood cells, and this method usually involves a prolonged incubation step (~18 h) [87,91]. A more rapid process involves targeted cell lysis [92]. This has been achieved by using saponin and deionised water to differentially lyse blood cells, followed by the separation and concentration of remaining bacterial cells with a custom-made microfluidic device [87]. This method results in almost all bacteria being concentrated in the final sample, with virtually no blood debris. The final product can then be used for downstream nucleic acid testing [87]. Boardman et al. described a similar method, focusing on concentrating lower bacterial loads (up to 100 CFU/mL) in a final 30 µL sample from an initial blood volume of 10 mL [88]. They applied the method to blood samples spiked with 100 CFU/mL *S. aureus*, followed by qPCR and reported a sensitivity and specificity of 97% and 96%, respectively [88]. While these results are encouraging, these studies are limited by their analysis of only single pathogens. In addition, more work is needed to determine if such devices can function efficiently with much lower starting sample volumes and bacterial loads < 100 CFU/mL. 

#### 3.2.3. Next-Generation Sequencing (NGS)

Another molecular approach for sepsis diagnosis is sequencing the 16S rRNA gene found in prokaryotes [93]. This method can identify sepsis pathogens with higher sensitivity than routine culture, even when using low blood volumes from neonatal patients [94,95]. Interestingly, it has also been shown to detect clinically significant sepsis pathogens in polymicrobial infections not identified by the routine method. Faria et al. demonstrated this in a case study that reported the presence of polymicrobial DNA in blood from three septic patients, indicating polymicrobial bacteraemia [96]. When the method was applied to clinical samples in a subsequent study using paired-end Illumina sequencing, they once again detected the presence of polymicrobial DNA [7]. A 2016 study by Decuypere and colleagues confirmed a similar finding, that blood culture completely missed detection of 10 out of 22 cases [8]. Even more striking is that over 40% of polymicrobial infections were not detected by routine methods [8]. These studies indicate that the true prevalence of polymicrobial sepsis is likely underestimated, and there might actually be a greater tendency for polymicrobial infections in sepsis than currently believed. Further epidemiological research into polymicrobial sepsis should employ sensitive methods such as 16S rRNA gene sequencing to investigate this.

While 16S rRNA gene sequencing has often been considered to be unfeasible for routine diagnostics due to traditional approaches having lengthy turnaround times and involving extensive bioinformatics analysis, these drawbacks can be improved in time with more automation, standardised DNA extraction techniques, and user-friendly computational tools [93]. Newer sequence-based analysis software tools utilising curated databases that are filtered to be more ‘medically relevant’ can also result in more accurate pathogen identification. Rip-seq (Pathogenomix Inc., Santa Cruz, CA, USA) for example, excludes non-informative or erroneous gene references from databases, resulting in rapid (5 min per analysis) and accurate identification of pathogens, even in polymicrobial samples. A recent study demonstrated that 16S rRNA gene sequencing using Illumina MiSeq coupled with Rip-Seq analysis was able to correctly identify pathogens with 58% concordance with blood culture results, including a polymicrobial case with *E. coli* and *S aureus* [97]. The discordant results were most likely due to sample preparation and issues with the assays, such as inhibition, interference from human DNA and the use of cell-free DNA, which has a short half-life [97]. A Breakthrough Device Designation has recently been granted by the FDA for the Patho-Seq assay (Pathogenomix Inc., Santa Cruz, CA, USA), which combines sequencing and Rip-Seq technologies for the identification of pathogens from different sample types (e.g., whole blood and CSF) and several clinical conditions, including polymicrobial sepsis. As this approval has only been announced at the beginning of 2022, there is no data available to validate its performance for sepsis diagnostics. 

Sequencing the fragments of genomic pathogen DNA found in plasma (cell-free DNA) is another application of NGS that has been described for sepsis diagnostics [98]. This method, often referred to as metagenomic NGS (mNGS), is particularly relevant for polymicrobial samples, as it uses an unbiased metagenomics approach to identify mixed genomes in a sample [99]. Its performance for the detection of bloodstream infections is adequate, with one study reporting sensitivity and specificity of 87.1% and 80.2%, respectively [100]. It has also been shown to outperform blood culture for the detection of fungi and difficult-to-culture bacteria, with Wu et al. reporting an 87% pathogen detection rate compared to 59% for blood culture [101]. The usefulness of mNGS, particularly in detecting polymicrobial infections, has also been demonstrated in other related infectious diseases, such as pneumonia [102,103]. However, as Xie et al. have shown, pathogen detection in plasma samples may not be as reliable as in other samples, such as bronchoalveolar lavage fluid, due to lower pathogen abundances in blood [102]. 

Currently, the Karius test, which can detect DNA from 1250 bacteria, viruses, fungi, and parasites, is the only commercially available mNGS assay for plasma [98]. It has been shown to detect missed polymicrobial cases and has also been shown to have shorter turnaround times (~48 h) compared to traditional culture methods [98,104,105]. However, the detection of microbial cell-free DNA in blood does not necessarily imply that the organism is pathogenic. While healthy blood has previously been considered sterile, it is now evident that cultivatable bacteria, likely originating from other microbiomes, such as the gut and oral cavity, can translocate into the bloodstream and can be isolated from healthy individuals [106,107,108,109]. Furthermore, the clinical impact of cell-free DNA sequencing on sepsis patient outcomes is unclear [104,110,111]. A study assessing clinical impact in paediatric patients with suspected sepsis showed that the method was helpful to clinicians in guiding patient care in 52.1% of cases and directly resulted in clinical management changes, such as narrowing antibiotics coverage, in 32.4% of cases [104]. The application of cell-free DNA sequencing for sepsis diagnosis is still emerging and requires additional retrospective and prospective studies to be carried out to further assess the clinical impact and determine its diagnostic value. 

### 3.3. Limitations of Molecular Technologies for Detecting Polymicrobial Sepsis

#### 3.3.1. Assay Inhibition

Many molecular techniques require nucleic acid amplification by PCR in their workflow [112]. However, some blood components, including haemoglobin, lactoferrin and immunoglobulin G, are known to inhibit PCR reactions [113]. Blood cultures also contain inhibitors, such as sodium polyanetholesulfonate, an anticoagulant that also mitigates the effect of antibiotic interference [113]. These inhibitors exert their effects by reducing DNA polymerase activity, interacting with primers and DNA templates, or quenching fluorescence [114,115]. Although diluting the DNA extract can reduce these inhibitory effects, this can lead to DNA loss [116], particularly in low biomass samples such as whole blood. The PCR master mix should therefore include an inhibitor-tolerant, thermostable DNA polymerase. Different polymerases demonstrate varying results in the presence of inhibitors [116,117]. For example, the sensitivities of *Taq* DNA and Ampli*Taq* Gold polymerases have been shown to be compromised in the presence of only 0.004% (*vol*/*vol*) blood in the PCR mixture, whereas the sensitivities of HotTub and *Thermus flavus* (*Tfl*) polymerases were not affected in the presence of 20% (*vol*/*vol*) blood [117]. Further, the use of inhibitor-tolerant DNA polymerase buffer systems can significantly diminish inhibitory effects when working with challenging samples [118]. The KAPA3G, TEMPase and PerfeCta Toughmix systems have been determined to be among the best-performing systems in a study that screened 16 systems in soil and sediment samples [118]. Similar comparisons are crucial to ensure the selection of the most appropriate system when carrying out sepsis studies on blood.

#### 3.3.2. Turnaround Time

As mentioned in the previous sections, PCR performs better on positive blood cultures than whole blood samples. Although the PCR itself may take only 1–2 h, the automated incubation step requires at least 12 h but can be up to 48 h for slower-growing Gram-positive bacteria, which increases the overall turnaround time [119]. One way to overcome this is by incorporating a shorter culture step. Moore et al. have integrated this into their PCR/pyrosequencing assay workflow [95]. They determined that an enrichment time of 5.8 ± 2.9 h was adequate to accurately identify 92% of confirmed blood culture cases [95]. Importantly, the final results were available approximately 16 h before Gram stain results, and 3 days before final phenotypic results were available [95]. In a subsequent study, the same group tested the analytical performance of this test on clinical samples. Following an 8 h blood culture enrichment step, the assay could identify approximately 91% of all positive cases, with 90.9% and 99.6% sensitivity and specificity, respectively [120]. The authors determined that some of the discrepant results they observed were associated with polymicrobial infections [120]. However, only 3 of the 99 samples included in the study were clinically confirmed to be polymicrobial based on the routine method, so further research on the effect of shorter culture on the detection of polymicrobial infection is required on a larger scale. In particular, more research is needed to determine how shorter culture affects the accurate detection of organisms present at much lower concentrations, and if detection is affected by polymicrobial scenarios that involve high/low titres of pathogen combinations. 

#### 3.3.3. Contamination

The high sensitivity exhibited by molecular tests makes them susceptible to false-positive results due to contamination, especially when analysing low biomass samples like blood [93]. Contamination may occur at the pre-analytical or sample processing stages. Meticulous sterile techniques during sample collection including the use of gloves and applying sufficient skin antiseptic can reduce contamination at the pre-analytical stage [121]. The frequency of isolating skin microbiota can also be reduced with initial-specimen diversion techniques (ISDT), which avoid culturing the initial blood sample by diverting initial blood to vacuum blood collection tubes for other testing (e.g., biochemistry) or to designated devices before collecting blood into blood culture bottles [122]. 

Clinically significant organisms, such as *E. coli* and *S. aureus*, are known to contaminate the environment and laboratory reagents, while others, such as *Enterococcus* spp. and *Proteus* spp. have been detected in sterile blood culture media [123]. Therefore, to determine whether isolated organisms are clinically significant and whether bacteraemia is truly polymicrobial, contaminating DNA must be removed or accounted for. There are two main ways to mitigate the effects of assay contamination [124]. Firstly, the inclusion of appropriate controls is crucial [124]. This includes negative controls (DNA extraction blank and no template amplification control), as well as positive controls during DNA extraction and amplification (e.g., titrations of DNA from known species), which allows for monitoring cross-contamination [124]. The second measure involves the treatment of potentially contaminated laboratory reagents [124]. For example, Stinson and colleagues found that PCR master mixes are a major source of bacterial DNA contamination and that this can be largely eliminated in low biomass samples by treatment with a double strand-specific DNase [125]. Many microbiome studies that have reported ‘true’ biological signals may in fact be erroneous due to their lack of controls. This risk needs to be minimised when working with blood, as signals from contaminating DNA can overshadow signals from DNA that is endogenous to the sample.

#### 3.3.4. Pathogen Enrichment

Another issue to overcome when applying molecular tools to blood is the high amounts of host DNA present in relation to microbial DNA [126]. This can be improved by incorporating host DNA depletion methods before DNA extraction to enrich microbes. The most widely used approach works on the principle of selectively lysing host cells and then removing host DNA by enzymatic digestion. For example, Nelson et al. demonstrated that host cells can be lysed by hypotonic lysis using saponin [127]. This is then followed by host DNA inactivation using an endonuclease such as benzonase [127]. When applied to whole blood, this method has been demonstrated to have minimal effects on the viability of remaining Gram-positive and Gram-negative bacteria [96]. 

Several commercially available kits work on the same principle and have been shown to outperform other methods, such as the removal of methylated host DNA [127,128]. One study that tested various kits on diabetic foot infection tissue samples reported that kits using the selective lysis method (QIAamp DNA microbiome kit and Zymo HostZERO microbial DNA kit) resulted in better microbial DNA enrichment than the methylated DNA removal method (NEBNext Microbiome DNA Enrichment kit). For example, the QIAamp kit reduced the host-to-bacterial DNA ratio (18S/16S rRNA gene ratio) by 32-fold and increased the bacterial DNA component by more than ten-fold, whereas NEBNext still led to a high 18S/16S rRNA gene ratio (0.701 ± 0.022) [128]. However, the MolYsis kits (Molzym, Bremen, Germany), which also utilise the selective lysis method, show conflicting data across studies. While shown to achieve up to 9580-fold bacterial DNA enrichment following whole genome sequencing on spiked prosthetic joint fluid [129], and effective bacterial DNA isolation from whole canine blood resulting in up to 87% detection rate [130], it has also been reported to be the worst performing of three methods (Sepsityper, MolYsis and centrifugation) in BacT/ALERT bottles, with only 50.5% of bottles correctly identified [131]. A comprehensive study comparing the different techniques on blood would provide insight into how to proceed for future sepsis studies utilising molecular microbiology tools. 

#### 3.3.5. Differentiating between Viable and Non-Viable Organisms

Another limitation of molecular methods is that there is no way to easily determine whether amplified or sequenced DNA originates from live or dead pathogens, leading to a possible overestimation of viable cell numbers if all cells are considered viable [93]. This is important to address when seeking to study the structure and function of a polymicrobial community. Unfortunately, this is an area where there has been little progress, and there are currently no reproducible standard methods. However, two main ways have shown varying success [132]. The first involves assessing the integrity of the bacterial cell membrane, as it is assumed that cells without intact membranes are not viable [132]. Incorporating viability dyes, such as propidium monoazide (PMA), into molecular assays is one of the more common approaches [132]. Askar et al. evaluated a PMA-PCR test for detecting pathogens from prosthetic joint infection samples [133]. The method exhibited a sensitivity and specificity of 79% and 89%, respectively, whereas culture had 50% sensitivity and 98% specificity [133]. However, a recent study, which incorporated PMA into 16S rRNA gene sequencing, found that this was only successful when applied to mock spike-in communities containing two organisms but could not accurately quantify viability in environmental samples with more complex communities [134]. This suggests that while the method is the most promising that is available, substantial optimisation will be required when applying to blood to account for factors that affect PMA performance, such as sample turbidity and biofilms [134].

The second method relies on analysing bacterial gene transcription, as this is one of the first levels of cellular response [132]. Running complementary RNA analyses such as RNA micro-array, 16S rRNA sequencing, or metatranscriptomics may provide a more complete picture of microbe viability. However, this approach is more complicated, as RNA is far less stable than DNA, leading to faster sample degradation especially if appropriate storage buffers and conditions are not used [132]. This can result in signals from low-titre organisms being lost prior to RNA being extracted from the sample.

## 4. Pathogenesis of Polymicrobial Sepsis

### 4.1. Polymicrobial Interactions

**Figure 2:**
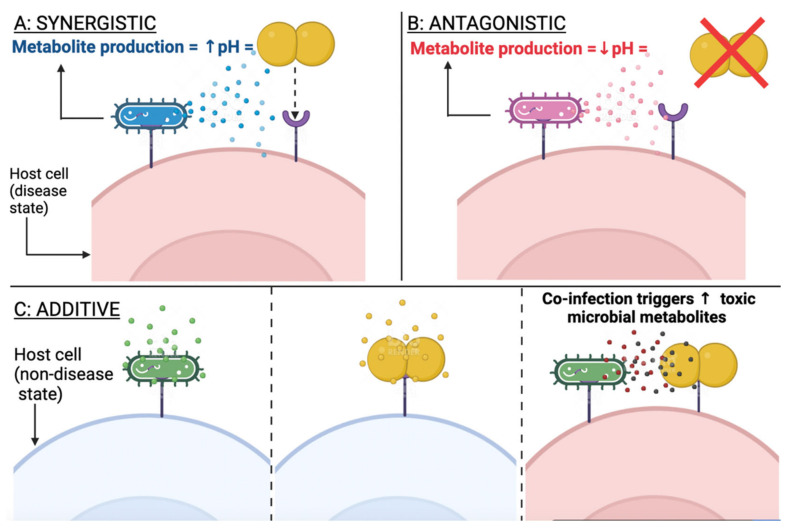
Examples of some interactions that can take place between microbes of different species (represented by different colours and shapes). (**A**)—colonisation of the first microbe compromises the host’s immune system, and creates an environment (e.g., increase in pH) that favours colonisation of the second microbe; (**B**)—colonisation of the first microbe creates an environment (e.g., product fermentation leading to pH decrease) that prevents colonisation of the second microbe; (**C**)—colonisation of two microbes on their own has no pathogenic effect, but cause disease when they co-infect.

Many host factors contribute to the progression of polymicrobial sepsis, but one aspect rarely considered is the effect of microbe–microbe interactions on pathogenesis. This is despite increasing evidence that polymicrobial interactions in other body sites, such as the lungs and wound infections, play an important role [18]. Figure 2 describes some of the polymicrobial interactions that may occur. During initial infection with an organism, the microbe can create an environment that allows another organism to colonise and co-infect [135]. The first microbe can also interact with the host and compromise its immune system to predispose it to secondary infections [135]. Conversely, the initial microbe can interact with the host in a way that prevents or reduces colonisation by a second microbe (antagonism) [135]. Polymicrobial interactions can also have an additive effect, whereby two microbes are non-pathogenic on their own but cause disease when they co-infect [135].

While many studies report worse patient outcomes associated with polymicrobial infections compared to monomicrobial infections, this is not always reflected in higher mortality rates. Recent studies have found no significant differences in mortality rates in polymicrobial *A. baumannii* bacteraemia compared to monomicrobial *A. baumannii* bacteraemia [23,24]. In fact, mortality rates were significantly lower in polymicrobial *A. baumannii* infections compared to monomicrobial infections in a recent systematic review [25]. Some authors have suggested this is because, in those scenarios, *A. baumannii* is only involved in colonisation rather than infection [25,136,137]. Therefore, many *A. baumannii* bacteraemias reported as polymicrobial may be monomicrobial infections with bacteria that are easier to eliminate with antimicrobials compared to *A. baumannii*, which is often multi-drug resistant [25,136,137]. However, another possible explanation is that interactions between co-pathogens affect their virulence, e.g., by increasing *A. baumannii* susceptibility to antimicrobials [138], resulting in less severe patient outcomes. 

There are well-documented accounts of polymicrobial interactions in diseases traditionally considered monomicrobial, such as urinary tract infections (UTIs) and lung infections [18]. In the lungs, one of the most studied bacterial interactions is between *Pseudomonas aeruginosa* and *S. aureus*. In several studies, *P. aeruginosa* is reported to outcompete *S. aureus* and a recent in vitro study reported reduced *S. aureus* susceptibility to vancomycin due to *P. aeruginosa* production of toxins, such as pyocyanin [32,139]. Lara et al. recently reported that enterococcal growth in mixed UTI communities can be promoted by other community members through folic acid production [140]. In an experimental catheter-associated UTI in mice, *E. faecalis* promoted co-infection with *Proteus mirabilis*, which exhibited increased cytotoxicity through increased urease activity, thus increasing tissue damage and the incidence of bacteraemia [141,142]. In addition, these interactions resulted in increased biomass and complexity of the biofilm structure, which led to the protection of both organisms from antibiotics, such as ceftriaxone [141]. Interestingly, in a study by Zheng and colleagues, enterococci resistance to tetracycline was found to be higher in polymicrobial bacteraemia compared to monomicrobial bacteraemia, indicating similar interactions may occur during polymicrobial sepsis [143]. 

We currently do not have any insight into the types of microbial interactions involved in sepsis, perhaps due to the propensity for polymicrobial infections only recently coming to light, combined with difficulties associated with detection [6]. One way to study such interactions is by implementing a multi-omics approach, which is increasingly being applied to study the host’s response to sepsis [144]. A complete omics approach would involve studying microbes on four levels [145]. Studying the metagenome (DNA level) would provide insight into all of the genes present [145]. The microbial transcriptome (RNA level) would reveal which cells are metabolically active and which genes are actually being expressed [145]. Studying the metaproteome would reveal which transcripts translate into proteins, giving us an even better idea of the microbes’ functionality [145]. Finally, the ‘endpoint’ of many of these studies is metabolomics, which informs on which bacterial metabolites are produced during infection [145]. Ultimately, these metabolites are involved with polymicrobial interactions, as metabolites produced by microbes alter the host environment and affect how other microbes respond [146]. For example, *Porphyromonas gingivalis* is able to persist in polymicrobial oral infections with *Streptococcus gordonii* due to the production of streptococcal 4-aminobenzoate/para-amino benzoic acid (pABA), which encourages the accumulation of *P. gingivalis* [147]. However, information from metabolomics would be meaningless without taxonomical information from metagenomic data [148]. In fact, the metagenome complements all omics research and should therefore be the first to be mined in the quest to understand polymicrobial sepsis [148].

### 4.2. Shotgun Metagenomics to Study the Microbial Metagenome

Shotgun metagenomics is a tool that can allow us to study the microbial metagenome. It involves the extraction of all DNA present in a sample, which is then sheared into small fragments and sequenced individually [149]. This untargeted sequencing approach leads to the generation of taxonomic information from conserved genes, such as the 16S rRNA gene, as well as a complete overview of the panoply of genomes present, including information on virulence, antimicrobial resistance and metabolic potential [69]. Theoretically, the identification of pathogens and antimicrobial susceptibility testing (AST) could be determined using the same test [69,150]. Barraud et al. demonstrated this by using shotgun metagenomics to correctly profile bacteria in urine samples from sepsis patients, while also detecting antimicrobial resistance (AMR) genes that corresponded with standard AST results [151]. Unfortunately, this process can take longer than the current routine blood culture method, and the extensive expertise in bioinformatics required for analysis makes it unsuitable for use in routine laboratories [151]. Further, detection of an AMR gene does not always translate to AMR expression [148]. However, this technology has contributed significantly to our understanding of the functional potential of microbial communities in diseases.

Metagenomics has been used to study differences in healthy and disease states to identify disease signatures. For example, it has been revealed that samples from periodontal disease patients have more genes associated with opportunistic traits or virulent traits, such as uptake of nutrients from lysed cells and LPS biosynthesis [152]. In contrast, healthy samples contained genes associated with more protective traits, such as fatty acid biosynthesis [152]. Similarly, metagenomics has been used to compare the vaginal microbial communities and their functions between preterm birth (PTB) and full-term birth (FTB) [153]. Feehily and colleagues reported the bacteria *Lactobacillus crispatus* was associated with FTB, suggesting a protective role, whereas *Sneathia amnii* and *Prevotella amnii*, which are associated with poor pregnancy outcomes, were higher in cases of PTB [153]. Further, the functional potential of the microbial gene pool differed significantly between PTB and FTB, with PTB being more associated with functions such as folic acid biosynthesis [153]. These studies demonstrate that metagenomics can be leveraged beyond taxonomy purposes to uncover functional gene profiles. Assessing bacterial gene profiles in terms of disease and disease outcomes can provide insights into the microbial contribution to pathogenesis, and may ultimately be useful for predicting patients at risk of developing a disease. This in turn may lead to the development of more targeted diagnostic and therapeutic tools.

The attention should then shift towards applying this technology to understand the mechanisms involved with polymicrobial interactions in sepsis. Shotgun sequencing technologies are readily available through Illumina, PacBio, Oxford Nanopore and Ion Torrent [154,155]. Future research could utilise these platforms to analyse clinical samples to determine: (1) if polymicrobial sepsis cases with the same co-pathogens share common functional gene profiles, and (2) if cases with the same disease outcomes share the same functional gene profiles. Findings from such work would provide novel insights into the microbial contribution to sepsis pathogenesis and could then be followed by bacterial and host transcriptomics, proteomics and metabolomics studies to identify the interacting metabolic pathways involved in disease. In addition to assisting with the development of novel sensitive and rapid culture-independent diagnostics, this would bring us a step closer to developing precision medicine in sepsis by complementing research into the host immune response signature, ultimately helping with identifying patients at higher risk of developing severe outcomes, and patients who are more likely to respond to pathogen-directed treatments.

## 5. Conclusions

Polymicrobial sepsis is associated with higher morbidity and mortality than monomicrobial sepsis. Usual care diagnostic modalities are suboptimal for polymicrobial detection, resulting in delayed and inaccurate diagnoses. Sensitive and rapid culture-independent microbiology diagnostic methods are urgently required. Limitations of current molecular microbiology technologies, including contamination and host DNA interference, should be addressed to create reliable alternative diagnostic tools.

The ultimate goal should be to optimise the accuracy and speed of methods for total pathogen detection and develop targeted diagnostic approaches for the most commonly associated polymicrobial organisms based on extensive epidemiology studies. Recent advances in omics technologies could help uncover pathogen–pathogen interactions involved in the pathogenesis of polymicrobial sepsis. Shotgun metagenomics generates information on potential microbe functionality and informs later transcriptomics, proteomics and metabolomics investigations. This could provide a more precise understanding of polymicrobial sepsis and inform the development of rapid molecular diagnostics to guide clinicians to improve patient management and reduce sepsis driven morbidity and mortality. 

## Figures and Tables

**Table 1:** Various definitions of polymicrobial bacteraemia used in the literature.

Definitions		Estimated Incidence of Polymicrobial Bacteraemia
1	Isolation of more than one pathogen from the same blood culture [11,20]	~6.7–11.4%
2	Isolation of more than one pathogen from one or more blood cultures within 48 h [3]	~4.4%
3	Isolation of more than one pathogen within 72 h [14]	~2%
4	Isolation of more than one pathogen from blood culture samples during an entire infectious episode [9]	~10.6–14%

**Table 2:** Risk factors for developing polymicrobial sepsis.

Population	Identified Risk Factors	Estimated Odds (Odds Ratio) of Developing Polymicrobial Compared to Monomicrobial Sepsis
Adults (hospitalised)	Foreign bodies [14]	2.3
	Recent invasive procedures [14]	3.6
Adults (community)	Biliary tract infections [28]	7.2
Adults (immunocompromised)	Neutropenia [15]	2.2
	Burn ward hospitalisation [26]	6.1
	ICU hospitalisation [26]	2.4
	Abdominal infections [15]	2.2–2.9
	Corticosteroid therapy [15]	1.5
Children (0–18 years)	Gastrointestinal (GI) pathologies [4]	2.4
	Presence of a central venous catheter [4]	11.3
Neonates (preterm)	Surgical intervention [9]	2.4
	Chronic GI pathology [3]	6.0
	Intubation [3]	2.8

## Data Availability

Not applicable.
